# MASDF-Net: A Multi-Attention Codec Network with Selective and Dynamic Fusion for Skin Lesion Segmentation

**DOI:** 10.3390/s24165372

**Published:** 2024-08-20

**Authors:** Jinghao Fu, Hongmin Deng

**Affiliations:** School of Electronics and Information Engineering, Sichuan University, Chengdu 610065, China; fujinghao@stu.scu.edu.cn

**Keywords:** skin lesion segmentation, vision transformer, skip connection, multi-scale

## Abstract

Automated segmentation algorithms for dermoscopic images serve as effective tools that assist dermatologists in clinical diagnosis. While existing deep learning-based skin lesion segmentation algorithms have achieved certain success, challenges remain in accurately delineating the boundaries of lesion regions in dermoscopic images with irregular shapes, blurry edges, and occlusions by artifacts. To address these issues, a multi-attention codec network with selective and dynamic fusion (MASDF-Net) is proposed for skin lesion segmentation in this study. In this network, we use the pyramid vision transformer as the encoder to model the long-range dependencies between features, and we innovatively designed three modules to further enhance the performance of the network. Specifically, the multi-attention fusion (MAF) module allows for attention to be focused on high-level features from various perspectives, thereby capturing more global contextual information. The selective information gathering (SIG) module improves the existing skip-connection structure by eliminating the redundant information in low-level features. The multi-scale cascade fusion (MSCF) module dynamically fuses features from different levels of the decoder part, further refining the segmentation boundaries. We conducted comprehensive experiments on the ISIC 2016, ISIC 2017, ISIC 2018, and PH2 datasets. The experimental results demonstrate the superiority of our approach over existing state-of-the-art methods.

## 1. Introduction

**Figure 1:**
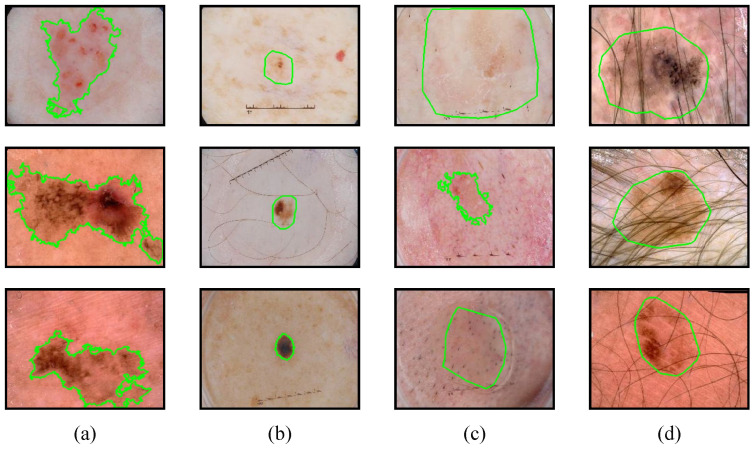
Some examples of challenging dermoscopic images: (**a**) skin lesions with an irregular shape, (**b**) skin lesions with too small sizes, (**c**) skin lesions with blurred boundaries, and (**d**) skin lesions obscured by hairs, where each green curve in the diagram represents the ground truth.

Skin cancer is one of the most common malignancies worldwide, contributing to 1.79% of the global disease burden, which is measured in disability-adjusted life years (DALYs) [1,2]. Melanoma is considered the deadliest form of skin cancer, and it accounts for 75% of skin cancer-related deaths [3]. Fortunately, if this type of skin cancer is detected at an early stage and treated effectively, its survival rate can be increased to over 99% [4]. Dermoscopy is one of the fundamental methods for diagnosing melanoma. However, the manual examination of dermoscopy images by dermatologists is often time consuming and requires a high level of skills and attention, and it is prone to operator bias. Computer-aided diagnosis (CAD) has become an effective tool for dermatologists in making decisions, especially when dealing with a large number of patients in a short period of time [5]. The segmentation of dermoscopic images is an important step in CAD. It distinguishes between normal and diseased regions within a patient’s skin, either through manual or other automated methods, preparing the groundwork for further diagnosis by dermatologists. Nevertheless, this task is quite challenging due to the complexity and variability of the lesion area (see Figure 1). Compared to other medical images, the lesions in dermatological images tend to have irregular shapes and uneven color distribution. Additionally, in the early stage of lesions, a low contrast between the lesion area and the surrounding skin results in blurred area boundaries. Furthermore, the lesion area is likely to be obscured by artifacts such as hairs, bubbles, etc. Therefore, the development of an automated and accurate skin lesion segmentation algorithm is important in clinical auxiliary diagnosis.

Traditional skin lesion segmentation algorithms like thresholding [6,7,8], region merging [9], and active contour models [10] are not only computationally complex, but also poorly robust; thus, they can hardly cope with complex skin-lesion scenarios. In contrast, deep learning models based on convolutional neural networks (CNNs) can not only learn the boundary features of skin lesion areas adaptively, but they can also outperform traditional solutions in terms of performance and accuracy. However, traditional CNN architectures were unable to achieve pixel-level predictions until the emergence of the fully convolutional network (FCN) [11]. The FCN replaced the fully connected layer in the final stage with a convolutional layer, enabling end-to-end, pixel-to-pixel training and effectively addressing the challenges in semantic segmentation. Due to the excellent performance of the FCN on image segmentation tasks, a large number of FCN-based networks were proposed. These include, for example, the representative asymmetric network DeepLabv3+ [12] and the symmetric networks SegNet [13] and U-Net [14]. Among them, U-Net is undoubtedly the most widely used network in the field of medical image segmentation. Many subsequent improvements for medical image segmentation are based on it. For example, Attention-UNet [15] introduced an attention-gating mechanism to U-Net to suppress irrelevant information. After-UNet [16] employed an axial fusion mechanism to effectively handle 3D medical image segmentation tasks while reducing the computational complexity of the self-attention in 3D space to some extent. ERDUnet [17] designed a differential regional attention mechanism that extracts high-level features from different regions of features at different stages, refining the segmentation boundaries. FD-Net [18] applies a compact-feature distillation block in the encoding stage, fully extracting features of different levels from the high-dimensional input. NFMPAtt-Unet [19], to address the complex structures and uncertainties in medical images, proposed a fuzzy C-means feature extraction module based on a neighborhood rough set. It utilized the concept of neighborhood rough sets for fuzzy C-means feature extraction, enhancing the model’s adaptability. While medical image segmentation methods based on U-Net have shown their superiority, they still face the following challenges due to the complexity of dermoscopy images: (1) The networks are usually constrained by the local properties inherent to convolutional operations, so it is not easy to capture the long-range dependencies [20]. In particular, when dealing with irregularly shaped or blurred boundary lesions, they tend to focus on local features, which could lead to inaccurate segmentation. (2) The existing methods typically incorporate only one or two types of attention mechanisms in the network, lacking comprehensive attention to the global contextual information from various perspectives. This could be detrimental when trying to segment lesions with irregular shapes or complex backgrounds. (3) The design of skip connections can potentially overemphasize low-level features while ignoring high-level semantic information, which could introduce noise or irrelevant details [21], especially in the presence of artifacts like hair and bubbles. (4) Continuous upsampling operations in the decoder phase inevitably lead to the loss of deep semantic information while restoring spatial positional information [22]. This could cause the model to potentially fail to accurately identify and segment the exact boundaries of skin lesions, especially when the lesion area is irregular or blurred.

In recent years, due to its powerful global modeling capability, Transformer [23] has been widely applied in the field of computer vision. For example, Zamir et al. proposed a Transformer with an encoder–decoder structure called Restormer [24], which was used for multi-scale local–global representation learning on high-resolution images and achieved state-of-the-art results in several image restoration tasks. Li et al., inspired by self-attention in Transformers, introduced a multi-head transposed cross-attention module in their framework, which demonstrated good performance in low-resolution image object detection tasks [25]. For saliency object detection tasks, the Visual Saliency Transformer [26] proposed a new label upsampling method within the Transformer framework, achieving top results on multiple datasets. Based on the excellent performance of Transformer models in various fields, recent research also attempted to integrate Transformers into medical image segmentation tasks. For instance, TransUNet [27] integrated Transformers into the U-Net architecture in a cascaded manner, achieving superior performance compared to previous methods in multi-organ segmentation and cardiac segmentation tasks. To simultaneously capture global dependencies and local spatial details, TransFuse [28] cleverly combined CNN and Vision Transformer in a parallel manner, achieving state-of-the-art results in tasks such as polyp and skin lesion segmentation. However, Vision Transformer and its variants typically faced issues with fixed input sizes and low locality inductive bias, often requiring high computational costs and large-scale training data [29]. Given the relatively small size of the skin lesion datasets and considering computational costs, we adopt pre-trained Pyramid Vision Transformer (PVT) [30] as the feature extraction backbone in the encoder phase, replacing the convolutional layers in the U-Net. This modification aims to effectively model the distant dependencies between lesion locations. The attention mechanism enables neural networks to focus on regions of interest and filter out irrelevant features. For instance, SE-Net [31] and ECA-Net [32] explicitly modeled interdependencies between channels to handle feature map channel-wise relationships. CBAM [33] enhanced network performance by concatenating channel attention and spatial attention. FcaNet [34] rethought global average pooling from a frequency-domain perspective and introduced a novel multi-spectral channel attention framework. However, the mentioned attention mechanisms only focus on one or two types of attention. To enhance the focus on global context information from multiple perspectives, we inserted the multi-attention fusion (MAF) module into the deep layers of the network. Our MAF module integrates multiple attention mechanisms. Initially, it generates a two-dimensional spatial attention map by combining global average pooling and global max pooling operations. Subsequently, a one-dimensional channel attention heat map is produced through a series of convolutions and multi-layer perceptron (MLP) operations. This heat map is capable of capturing long-range dependencies and enhancing the spatial features. Ultimately, this attention information is fused with the input features, thereby strengthening the network’s focus on deep global contextual information and achieving the preliminary exploration of the lesion area. Due to the semantic disparity between low-level features in the encoding phase and high-level features in the decoding phase, traditional skip connections introduce irrelevant noise. To mitigate the impact of irrelevant noise, subsequent approaches have introduced various improvements to the skip connections. For instance, CPFNet [35] incorporated a global pyramid guidance (GPG) module to fuse context information of different scales with features from the decoder stage, aiming to reconstruct the skip connections. However, this method solely relied on convolutional operations for local feature fusion, lacking the modeling of remote dependencies among features at different scales. Attention U-Net [15] and CA-Net [36] implicitly suppressed the influence of irrelevant background regions using attention gate units. However, this method is still influenced by the inherent local properties of convolution operations, resulting in a lack of ability to establish long-range information interaction among pixels at the same spatial position. UCTransNet [21] incorporated the Transformer into skip connections and utilized the CTrans module for collaborative learning rather than independent connections, effectively fusing multi-scale channel features that may exhibit scale-semantic discrepancies. Although this approach achieved some effectiveness, it came with a substantial increase in parameter count and computational demand. Additionally, due to the complexity of its modules, it was challenging to integrate it into existing encoder–decoder networks. Based on the issues presented by the aforementioned methods, we intricately designed the selective information gathering (SIG) module using cross-attention to achieve more efficient skip connections. Our SIG module, under the guidance of the cross-attention mechanism, allows low-level features to attend to the abundant semantic information in high-level features. This mechanism enables the model to selectively aggregate information during the skip-connection process, thereby reducing irrelevant noise caused by artifacts such as hair and bubbles, and enhancing the accuracy and robustness of the segmentation. Between different levels in the decoder stage, the feature maps encompass distinct contextual information: lower-resolution features carry more semantic content, while higher-resolution features contain greater positional and boundary information. Wu et al. [37] noted the significant contribution of low-resolution high-level features to the network’s performance. However, in the decoder stage, the upsampling of high-level features inevitably results in the loss of semantic information. Thus, we devised the multi-scale cascade fusion (MSCF) module for the final stage of our model. Our MSCF module consists of a cascade module and a scale-aware module. The cascade module processes features in a sequential manner, with the output of one layer serving as the input for the subsequent layer. This step-by-step refinement allows the network to iteratively improve the segmentation results, building upon each previous stage to enhance boundary localization. The scale-aware module does not merely concatenate or sum features from different layers; instead, it dynamically adjusts the contribution of each layer based on the specific context of the image. This enables the network to focus on the most relevant features for boundary refinement. By integrating features from various layers, the MSCF module enhances the contextual information used for decision-making, which is particularly beneficial when dealing with complex scenarios featuring blurred or obscured boundaries.

In summary, the main contributions of this paper are as follows:

1. We propose a novel multi-attention encoder–decoder network with selective and dynamic fusion named MASDF-Net, which effectively addresses the challenges of segmenting the skin lesions with irregular shapes, blurry boundaries, and noise interference.
2. We design the MAF module based on multi-attention mechanisms, aiming to enhance the network’s focus on global context information at deeper layers from multiple attention perspectives.
3. For the enhancement of skip connections in the U-shaped network, we design the SIG module based on cross-attention. This module interacts in a learnable manner to propagate rich positional information from low-level features and semantic information from high-level features, alleviating the semantic gap between the encoder and decoder.
4. We design the MSCF module to dynamically fuse features of different scales in the decoder stage, leading to improve the final segmentation results.

We conduct extensive experiments on four publicly available skin lesion datasets. The experimental results consistently demonstrate that our approach outperforms the state-of-the-art methods in terms of both performances and generalization capabilities. The remaining sections of this paper are organized as follows: Section 2 reviews the works related to our research. Section 3 provides a detailed exposition of our model and the key modules designed for this model. Section 4 presents our experimental results along with comparative analyses. Finally, in Section 5, we conclude our work.

## 2. Related Work

This section reviews some of the work closely related to our study, including U-Net and its variants, Vision Transformer and Transformer, in medical image segmentation.

### 2.1. U-Net and Its Variants

Since its introduction in 2015, U-Net has experienced explosive growth in the applications of medical image segmentation [38]. The basic structure of U-Net consists of an encoder and a decoder. The encoder, similar to a convolutional network, typically consists of multiple convolutional and pooling layers, responsible for extracting high-level semantic features from the input image. The decoder is responsible for remapping the abstract semantic features extracted by the encoder back to the input image space and restoring pixel-level detail information. Unlike FCN, the decoder of U-Net utilizes skip connections to fuse the features from both encoder and decoder, thereby preserving more details and boundary information. The current mainstream medical image segmentation networks still follow the idea of U-Net, by adding an attention mechanism and incorporating appropriate multi-scale contextual techniques to improve the segmentation performance of the model. For example, Attention-UNet [15] introduced attention gates to emphasize salient features and suppress irrelevant information during the skip-connection process, which achieved the goal of improving the accuracy of model segmentation with less computational overhead. UNet++ [39] was influenced by the nested skip connections in DenseNet [40] and redesigned the skip paths to add more dense connections to fuse different levels of semantic information. CE-Net captured more advanced features and spatial information through a context extractor module consisting of dense atrous convolution (DAC) block and residual multi-kernel pooling (RMP) block [41]. CPFNet was designed with global pyramid guidance (GPG) module and scale-aware pyramid fusion (SAPF) module to fuse global and multi-scale contextual information [35]. MSCA-Net proposed the scale-aware deep supervision (SADS) module, which performed deep supervision in a hierarchical and iterative form [42]. However, since these networks were implemented based on convolutional operations, they usually had limitations when establishing remote dependencies.

### 2.2. Vision Transformer

In the field of natural language processing (NLP), because the self attention mechanism in Transformers [23] could dynamically establish the long-term dependency of input sequences, it has become the preferred model to solve NLP problems. Based on the great success of Transformers in the field of NLP, some scholars also tried to introduce the idea of self-attention into the field of computer vision. Wang et al. [20] first introduced the self-attention mechanism into CV and obtained the remote dependency between pixels through the non-local operation. Dosovitskiy et al. [43] were the first to apply a pure Transformer model to image classification tasks and achieved SOTA performance with large-scale data pre-training. DeiT [44] improved the training efficiency of ViT by using an efficient distillation strategy that allowed ViT to converge well on the smaller ImageNet-1K dataset. SETR [45] rethought the semantic segmentation paradigm by replacing the traditional encoder with Transformers and treating the semantic segmentation problem as a sequence-to-sequence prediction problem. Swin Transformer [46] utilized the idea of shifted windows to effectively reduce computational cost and achieved good performance in multiple downstream tasks without affecting information transfer. Wang et al. [30] proposed pyramid vision transformer (PVT), which used a gradually shrinking pyramid to reduce the computational effort. Benefiting from its pyramid structure, PVT overcame the difficulty of adapting the Transformer to various intensive prediction tasks, and directly replaced the CNN-based backbone. In PVT v2 [47], Wang et al. had adopted the technique of overlapping patch embedding (OPE) for image encoding, which ensured the preservation of local continuity among adjacent patches. Concurrently, they had substituted the attention layers present in the original PVT with linear spatial reduction attention (LSRA), an approach designed to attain a computational complexity analogous to that of convolutional neural networks (CNNs). Additionally, they had integrated the convolutional feed-forward network (CFFN), utilizing a zero-padding convolutional layer to ascertain local continuity within the input tensor, thereby enhancing the network’s adaptability to images of diverse resolutions.

### 2.3. Transformer in Medical Image Segmentation

TransUnet [27] pioneered the introduction of Transformer to medical image segmentation by encoding tokenized image patches from CNN feature maps as input sequences for extracting global context, which cleverly accomplishes the combination of CNN and Transformer. Due to the excellent performance of the Swin Transformer, Swin-UNet [48] replaced the convolutional layer in the U-shaped network with a pure Transformer structure. Lin et al. [49] argued that existing Transformer-based U-Net models overlook the pixel-level structural features within each patch. To alleviate these problems, they combined the benefits of a hierarchical Swin Transformer into a standard U-Net for the first time. However, these methods simply single out the Transformer as an encoder or decoder. In order to simultaneously capture low-level spatial features and improve the modeling efficiency of global context, TransFuse [28] and X-Net [50] combined Transformer and CNN in a dual-branch manner during the encoding stages to address this challenge. However, their approach to integrating information from different branches was achieved solely through straightforward shortcuts and channel concatenation, leading to an incompatibility between global and local features. Zhu et al. [51] proposed a brain tumor segmentation method based on deep semantic segmentation and edge detection, utilizing Transformers to extract semantic features for brain tumor segmentation, and CNNs to extract edge features. Subsequently, they employed multi-feature inference block (MFIB) to achieve effective feature fusion. Similarly, during the encoding and decoding stages, TC-Net [52] simultaneously integrated Transformer and CNN in parallel, effectively combining features obtained from two branches through a locality-aware and long-range dependency concatenation strategy. Although these strategies integrating CNN and Transformer in parallel have achieved some success, the dual-branch approach inevitably leads to parameter inflation. Moreover, existing methods have not proposed an effective solution for the efficient fusion of features extracted by CNN and Transformer. Therefore, in our proposed MASDF-Net, we employed PVT v2, which simultaneously considers the strengths of CNN and Transformer, as the feature extraction backbone in the encoder phase. This choice aims to efficiently capture both global and local information in skin lesion images.

## 3. Methodology

In this section, we introduce the overall architecture of MASDF-Net and our three key modules specially designed for this model: multi-attention fusion (MAF) module, selective information gathering (SIG) module and multi-scale cascade fusion (MSCF) module.

### 3.1. Network Architecture

**Figure 2:**
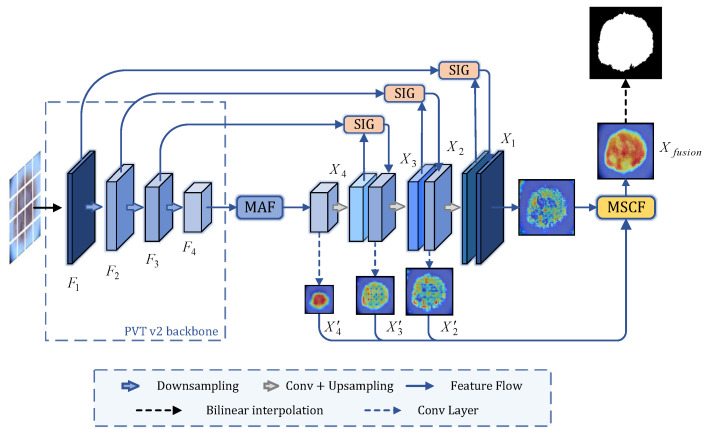
The overall framework of the proposed MASDF-Net. To begin with, we feed the dermatoscopic images into the PVT v2 backbone, obtaining features from different stages. The deepest-layer feature undergoes the multi-attention fusion (MAF) module to extract additional global contextual information. The selective information gathering (SIG) module is utilized to enhance skip connections and mitigate the introduction of irrelevant noise. The multi-scale cascade fusion (MSCF) module is employed to merge features from various stages (as visualized in the diagram).

The proposed MASDF-Net is illustrated by Figure 2, which consists of PVT v2 as the feature extraction backbone network and combines three proposed modules to accomplish the segmentation task. Specifically, given an input image $I\in R^{H\times W\times C}$, where *H*, *W*, and *C* denote the height, width, and number of channels of the image, respectively. After passing through PVT v2, we can obtain feature maps of four stages, and we denote the output features of ${\mathrm{stage}}_{i}$ as $F_{i}\in R^{\frac{H}{2^{i+1}}\times \frac{W}{2^{i+1}}\times C_{i}}$, where $C_{i}\in \left\{64,128,320,512\right\}$, i=1,2,3,4. Then we input $F_{4}$ into MAF to obtain $X_{4}$ for more contextual information. $F_{3}$, $F_{2}$ and $F_{1}$ effectively perform skip connections with the corresponding features on the decoding side through SIG, resulting in $X_{3}$, $X_{2}$, and $X_{1}$. We pass $X_{2}$, $X_{3}$, and $X_{4}$ through a series of convolutional layers to adjust their channel dimensions to 64, resulting in $X_{2}^{\prime }$, $X_{3}^{\prime }$, and $X_{4}^{\prime }$. Subsequently, they are combined with $X_{1}$ and fed into the MSCF module to dynamically fuse information from different scales of the decoder, enabling the more efficient segmentation of the skin lesion area.

### 3.2. Multi-Attention Fusion Module

**Figure 3:**
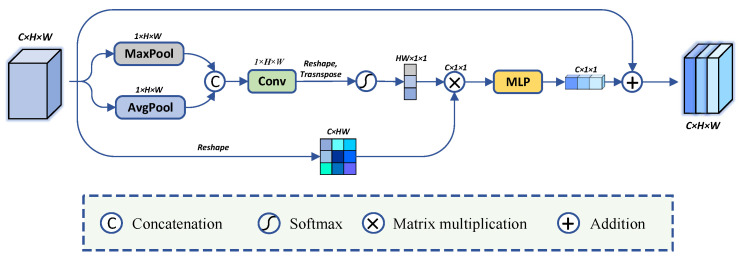
The details of multi-attention fusion (MAF) module.

Attention mechanisms can be used to enhance valid features and suppress irrelevant information. However, existing methods usually focused on only one or two attention mechanisms [31,33,53]. In order to be able to extract more contextual information in the deeper layers of the network, inspired by GCNet and CBAM, we propose multi-attention fusion (MAF) module. As shown in Figure 3, the input features generate a two-dimensional spatial attention map through two parallel pooling operations and a 1×1 convolutional layer. It is subsequently transposed and matrix multiplied with the input features (similar to a simplified non-local operation [20,53]) and fed into a multi-layer perceptron (MLP). Here, we then obtain a one-dimensional channel attention map that captures both long-local dependencies and enhanced spatial features. Finally, we fuse it with the input features to enhance the global context information. We set the input feature map as *X*, and the output feature map as *Y*. The specific steps of MAF can be summarized as follows:

(1) $$S_{a}\left(X\right)=Softmax\left({\left(f^{1\times 1}\left(\left[P_{avg}\left(X\right);P_{max}\left(X\right)\right]\right)\right)}^{T}\right)$$

(2) $$Y=MLP(X⊗S_{a}\left(X\right))+X$$

where $S_{a}\left(\cdot \right)$ is the spatial attention operation. $f^{1\times 1}$ represents a convolution operation with the filter size of 1×1. $P_{avg}\left(\cdot \right)$ and $P_{max}\left(\cdot \right)$ represent global average pooling and global maximum pooling, respectively. ⊗ denotes matrix multiplication. *T* means matrix transpose operation. MLP(·) consists of two fully-connected layers with a ReLU non-linearity and normalization layer.

### 3.3. Selective Information Gathering Module

**Figure 4:**
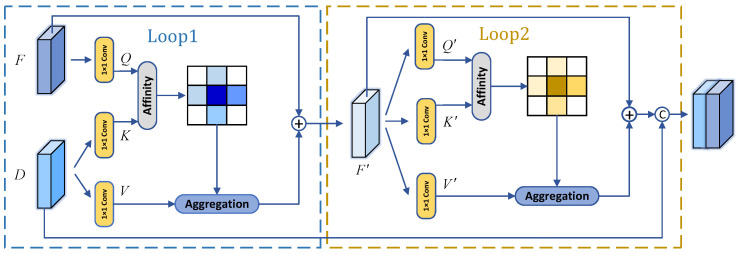
The structure of selective information gathering (SIG) module. We employ two loops to indirectly enable low-level features to attend to the comprehensive contextual information in high-level features.

In a U-shaped network, the encoder part usually leads to the loss of position information due to continuous downsampling, and the design of skip connections can enrich the spatial details by fusing the position information of low-level features with the semantic information of high-level features. However, due to the semantic differences between the encoder and decoder, simple skip connections can introduce irrelevant noise and ambiguity. In order to pay attention to the rich semantic information in high-level features and the details in low-level features simultaneously, we design a selective information gathering module based on cross attention as shown in Figure 4.

It is worth mentioning that we use criss-cross attention in [54] to improve the computational efficiency unlike the conventional non-local block. Given two symmetric feature maps *F* and *D* for the encoding and decoding stages, where $\left\{F,D\right\}\in R^{C\times W\times H}$. We use 1×1 convolution to generate a feature map *Q* on *F* and feature maps *K* and *V* on *D*. It can be expressed as $\left\{Q,K\right\}\in R^{C^{\prime }\times W\times H}$ and $V\in R^{C\times W\times H}$, where $C^{\prime }$ is the number of channels after dimensionality reduction of *C*. We perform affinity operation on *K* and *Q* to obtain $B\in R^{\left(H+W-1)\times (W\times H\right)}$. The attention map $A\in R^{\left(H+W-1)\times (W\times H\right)}$ is obtained by feeding *B* into the softmax layer.

The affinity operation works as follows:

(3) $$b_{i,u}=Q_{u}\Omega_{i,u}^{T}$$

where $b_{i,u}\in B$ refers to the degree of correlation between $Q_{u}$ and $\Omega_{i,u}$. $Q_{u}\in R^{C^{\prime }}$ represents each spatial position *u* in *Q*, and $\Omega_{u}\in R^{\left(H+W-1\right)\times C^{\prime }}$ represents the feature vectors in *K* that are in the same row and column of position *u*. $\Omega_{i,u}\in R^{C^{\prime }}$ is the *i*-th element of $\Omega_{u}$ and $i=[1,\ldots ,|\Omega_{u}|]$. Similarly, we obtain the feature vector $\Phi_{u}\in R^{(H+W-1)\times C}$ from *V* that is in the same row and column as the position *u*. Subsequently, we construct the aggregation operation, as shown below:

(4) $$F_{u}^{\prime }=\sum_{i\in |\Phi_{u}|}A_{i,u}\Phi_{i,u}+F_{u}$$

where $F_{u}^{\prime }$ is the feature vector of the output feature map $F^{\prime }$ at position *u*. Since criss-cross attention only enables each pixel point in *F* to collect information from the corresponding horizontal and vertical positions in *D*, we set up two loops to make *F* indirectly obtain the complete contextual information in *D* inspired by [54]. Finally, we let it concatenate with *D* on the channel to complete a more efficient skip-connection operation.

### 3.4. Multi-Scale Cascade Fusion Module

**Figure 5:**
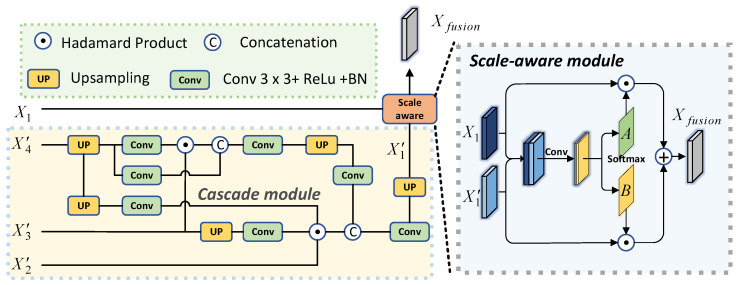
Details of the MSCF module, which consists of a cascade module and a scale-aware module.

In order to integrate different levels of features more effectively, we propose a multi-scale cascade fusion (MSCF) module consisting of a cascade module and scale-aware module, as shown in Figure 5. The $X_{1}\in R^{\frac{H}{4}\times \frac{W}{4}\times 64}$ has a high resolution and contains detailed spatial location information. For $X_{2}$, $X_{3}$ and $X_{4}$, which have lower resolution but contain more semantic information, we first adjust their channel counts by a series of convolutional units to obtain $X_{i}^{\prime }\in R^{\frac{H}{2^{i+1}}\times \frac{W}{2^{i+1}}\times 64}$, where i∈{2,3,4}. We utilize the most popular recent practices [37,55,56,57] to finish the feature fusion of $X_{2}^{\prime }∼X_{4}^{\prime }$. We define the output of the cascade module as $X_{1}^{\prime }=CM\left(X_{2}^{\prime },X_{3}^{\prime },X_{4}^{\prime }\right)$. Subsequently, in order to dynamically balance the weights between different scales, we introduce a scale-aware module [35]. Specifically, we concatenate $X_{1}^{\prime }$ and $X_{1}$ and feed them into the convolutional and softmax layers to obtain the spatial pixel-level maps *A* and *B*. The final output is obtained by weighted summation of the features at two different scales:

(5) $$X_{fusion}=X_{1}⊙A+X_{1}^{\prime }⊙B$$

where $X_{fusion}$ represents the fused feature map and the ⊙ represents the Hadamard product.

## 4. Experiments

### 4.1. Datasets

In order to compare the performance of our model with those of state-of-the-art methods, we conduct extensive experiments on four public skin injury datasets, including ISIC 2016 [58], ISIC 2017 [59], ISIC 2018 [60] and PH2 [61]. ISIC 2016, ISIC 2017 and ISIC 2018 were provided by the international skin imaging collaboration (ISIC) archive, and the PH2 database was created by a collaboration between the Universidade do Porto, TÃ©cnico Lisboa, and the Dermatology service of Hospital Pedro Hispano in Matosinhos, Portugal. Based on recent relevant work [42,62,63], four datasets are split as follows:

The ISIC 2016 dataset contains 1297 dermoscopic lesion images in JPEG format together with their ground truth (binary mask images) in PNG format, where 900 images are used for training and 379 images are used for testing.

The ISIC 2017 is a scaled-up dataset, providing 2000 training sets, 150 validation sets and 600 test sets.

The ISIC 2018 dataset consists of 2594 RGB images and the corresponding ground truth. In our experiments, it is randomly divided into training (70%), validation (10%) and test sets (20%).

PH2 is a small dataset containing only 200 dermoscopic images; in this paper it is used to evaluate the generalization ability of the model.

### 4.2. Loss Function

Skin lesion image segmentation is a typical pixel-level binary classification task: skin lesion and background. In this work, we combine the weighted intersection over union (IoU) loss and the weighted binary cross entropy (BCE) loss as our loss function [64]. The total loss function can be formulated as:

(6) $$L_{\mathrm{total}}=L_{\mathrm{IoU}}^{w}\left(P,G\right)+L_{\mathrm{BCE}}^{w}\left(P,G\right)$$

where $L_{\mathrm{IoU}}^{w}\left(\cdot \right)$ and $L_{\mathrm{BCE}}^{w}\left(\cdot \right)$ denote weighted IoU loss and weighted BCE loss, respectively, G represents the ground truth, and P represents the segmentation result.

### 4.3. Implementation Details

The proposed MASDF-Net is implemented in Pytorch 1.13.0 and an NVIDIA GeForce RTX 3070 graphic card (NVIDIA, Santa Clara, CA, USA) is utilized to accelerate the computation. We use the Adam optimizer for end-to-end training. The learning rate is set to 1 × $10^{-4}$, the batch size is set to 16, the max number of training iterations is 100, and the model with the highest Jaccard index score on the validation set is used to evaluate the performance of the network on the test set. In addition, we resize all the images to 224 × 224 and subject them to data augmentation operations such as random rotation, horizontal inversion, and color jittering before inputting them into the model.

### 4.4. Evaluation Metrics

Five widely approved evaluation metrics [65,66,67], are used by us to assess segmentation performance, including the Jaccard index (JI), Dice Score Coefficient (DSC), Accuracy (ACC), Sensitivity (SE) and Specificity (SP). The indicators are calculated as follows:

(7) $$JI=\frac{TP}{TP+FN+FP}$$

(8) $$DSC=\frac{2\cdot TP}{2\cdot TP+FN+FP}$$

(9) $$ACC=\frac{TP+TN}{TP+TN+FN+FP}$$

(10) $$SE=\frac{TP}{TP+FN}$$

(11) $$SP=\frac{TN}{TN+FP}$$

where TP, TN, FP, and FN represent true positive, true negative, false positive, and false negative, respectively [68].

### 4.5. Comparison with Several Existing Methods

In this section, we compare the proposed MASDF-Net with 10 state-of-the-art medical image segmentation networks including U-Net [14], AttU-Net [15], Deeplabv3+ [12], CE-Net [41], CPFNet [35], MSCA-Net [42], TransFuse [28], Swin-Unet [48], UCTransNet and Polyp-PVT [57]. The first six models were based on pure CNN architectures, while the last four models incorporated the Transformer in their network design. To ensure the fairness of the experiments, all networks are retrained under the same experimental conditions. Furthermore, to comply with the requirements of the ISIC challenge, we adopted JI as the primary metric for evaluating network segmentation performance, followed by DSC, ACC, SE, and SP in descending order of importance [42,63].

#### 4.5.1. Results on ISIC 2016, ISIC 2017 and ISIC 2018

**Quantitative Analysis:** As shown in Table 1, the proposed MASDF-Net shows the best segmentation performance on all three datasets. Benefiting from the formidable feature extraction capacity of the ResNet34 backbone network and the persistent integration of multi-scale features facilitated by the multi-scale bridge (MSB) module, MSCA-Net stands out as the most advanced network among all CNN-based approaches in terms of performance. Compared to MSCA-Net, the proposed MASDF-Net achieves improvements of 0.94%, 2.53%, and 1.32% in JI on the ISIC 2016, ISIC 2017, and ISIC 2018 datasets, respectively. Furthermore, our MASDF-Net only experiences a modest increase of 3.62% in parameter count compared to MSCA-Net, while demonstrating a substantial reduction of 55.10% in FLOPs (G). TransFuse was consistently the existing best-performing network for skin lesion segmentation tasks, especially on the ISIC 2017 dataset. This achievement was likely attributed to its integration of both CNN and Transformer as encoders. However, in this study, our model shows improvements over TransFuse in terms of JI, DSC, ACC on the ISIC2017 dataset, with increases of 1.4%, 1.05%, and 0.37%, respectively. Polyp-PVT was a network specifically designed for polyp segmentation, which also utilized PVT v2 as the feature extraction backbone network. Compared to Polyp-PVT, our model achieves improvements of 0.79%, 3.49%, and 1.22% in JI on the ISIC 2016, ISIC 2017, and ISIC 2018 datasets, respectively. This validates the effectiveness of our proposed MAF, SIG, and MSCF modules. Furthermore, according to Table 1 and Table 2, it is evident that there is a performance gap when segmenting challenging samples, compared to the average obtained in all cases. However, our method has an almost minimal gap. Of course, the Swin-Unet exhibits an even smaller performance loss due to its originally lower performance in Table 1. Therefore, the above results indicate that our model has a more pronounced advantage when dealing with challenging samples compared to other models.

**Figure 6:**
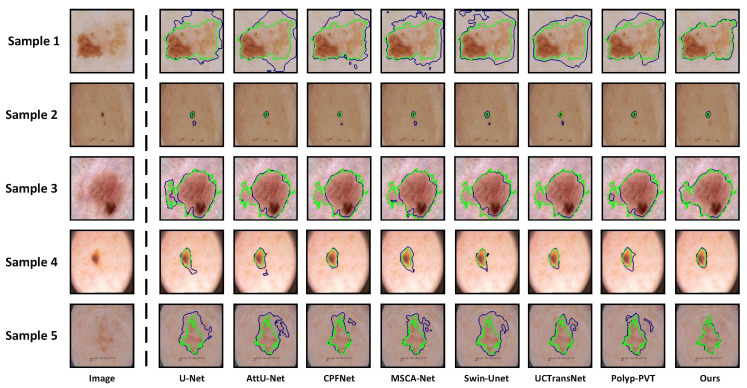
Segmentation results of lesions with irregular shapes and sizes. Sample 1 is from ISIC 2016, samples 2 and 3 are from ISIC 2017, and samples 4 and 5 are from ISIC 2018. The green curves represent ground truths, and the blue curves represent the predictions from different networks.

**Figure 7:**
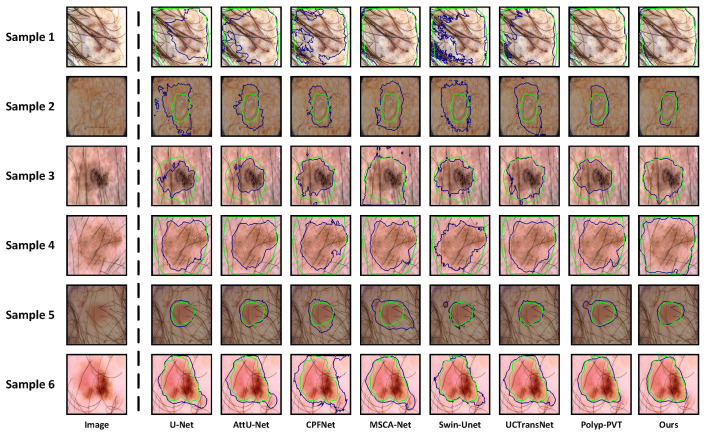
Segmentation results of lesions with hair interference, where sample 1 is from ISIC 2016, samples 2, 3 and 4 are from ISIC 2017, and samples 5 and 6 are from ISIC 2018. The green curves represent ground truths, and the blue curves represent the predictions from different networks.

**Figure 8:**
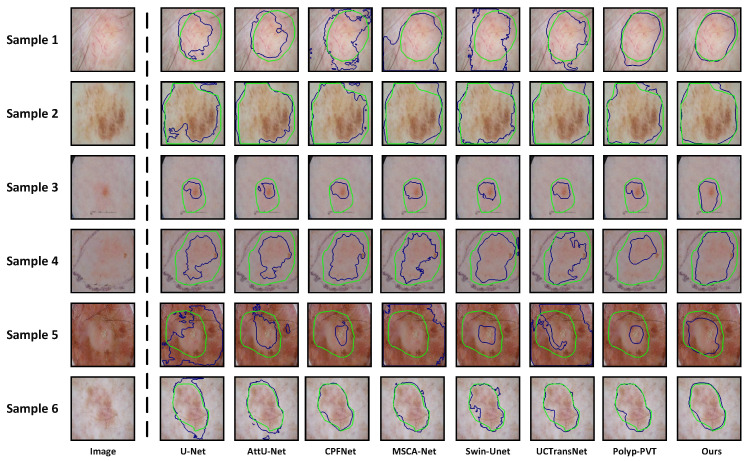
Segmentation results of lesions with blurred boundaries. Samples 1 and 2 are from ISIC 2016, samples 3 and 4 are from ISIC 2017, and samples 5 and 6 are from ISIC 2018. The green curves represent ground truths, and the blue curves represent the predictions from different networks.

**Qualitative Analysis:** On the ISIC 2016, ISIC 2017, and ISIC 2018 datasets, we provide the visualized segmentation results of some models on challenging samples, including lesions with irregular shapes and sizes, lesions obscured by hair, and cases with blurred boundaries. Figure 6 displays the results of some models on lesions with irregular shapes and sizes. We can observe that for samples 1, 3, and 5, which have more complex boundaries, existing models struggle to accurately segment the true lesion regions. On the other hand, for samples 2 and 4, which are smaller lesions, some models tend to misclassify the spots on the surrounding normal skins as lesions. The reasons behind these issues lie in the insufficient capability of these models to extract multi-scale information. However, due to the dynamic feature fusion ability of MSCF, our model maintains excellent segmentation performance even when handling lesions with irregular shapes and sizes. The interference of hair in dermoscopic images can severely affect the accurate segmentation of skin lesions. As shown in Figure 7, U-Net, AttU-Net, CPFNet, Swin-unet, and UCTransNet exhibit significant limitations in segmentation performance under such extreme conditions. Although MSCA-Net and Polyp-PVT show some improvement in performance, there is still a noticeable gap compared to the Ground Truth. In contrast, our model’s inclusion of SIG in the skip-connection process effectively reduces irrelevant noise interference, enabling the more accurate segmentation of lesion boundaries even in the presence of hair interference. The low contrast between the lesion area and the surrounding skin, leading to blurred boundaries, is the most significant challenge we face in skin lesion segmentation. Even experienced dermatologists find it difficult to accurately delineate the lesion area. From Figure 8, we can observe that some existing models are constrained by their limited ability to extract global context information, thus making it difficult to effectively segment lesion regions in low contrast environments. Due to the powerful global context information extraction capability of MAF module, our model can perceive subtle pixel variations, allowing for accurate lesion segmentation. From the above analysis, it is evident that our model exhibits clear advantages when dealing with some quite challenging dermoscopic images, which often pose difficulties for dermatologists in clinical diagnosis.

#### 4.5.2. Cross-Dataset Testing

**Figure 9:**
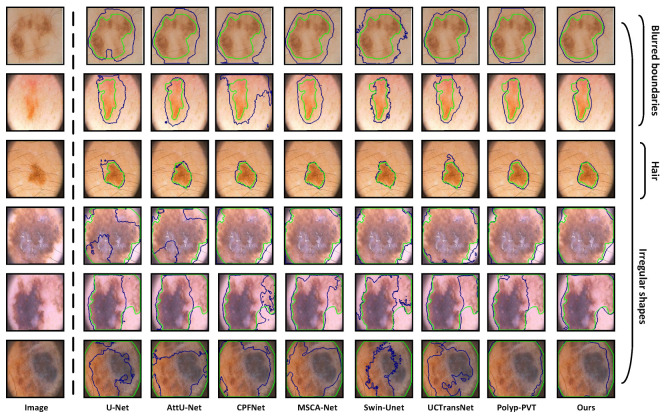
Qualitative segmentation results on the PH2 dataset. The green lines represent ground truth, and the blue lines represent the predictions from different networks.

To better evaluate the robustness and generalizability of our model, we conducted cross-dataset testing on the ISIC 2018 and PH2 datasets. Specifically, we tested our model trained on the ISIC 2018 dataset using the PH2 dataset. The quantitative results are presented in Table 3, where the data indicate that our model outperforms other state-of-the-art models in four crucial metrics, with our JI (84.64%), DSC (91.38%), ACC (95.06%) and SP (91.90%), respectively. In Figure 9, we also give the visualized segmentation results of some challenging samples from the PH2 dataset. However, due to the limitations in model generalization of existing methods, there is still a certain gap between the segmentation results and the ground truth, even for this relatively simple task. Thanks to the PVT v2 backbone network, which was pre-trained on large-scale datasets, our model boasts a strong generalization ability. Moreover, with the MAF module’s further extraction of global context information, the SIG module’s suppression of irrelevant noise, and the dynamic fusion of multi-scale features by the MSCF module, our model maintains robust segmentation performance even when facing cross-dataset testing.

### 4.6. Ablation Study

To validate the effectiveness of the proposed key modules within our MASDF-Net, we conducted a series of ablation experiments on the ISIC 2018 dataset. We primarily compared the following models:

**Baseline:** consisting of a U-Net with PVT v2 as the encoder.

**Model 1:** incorporating MAF module based on the baseline.

**Model 2:** incorporating SIG module based on the baseline.

**Model 3:** incorporating MSCF module based on the baseline.

**Model 4:** incorporating the SIG and MAF modules based on the baseline.

**Model 5:** incorporating the MSCF and MAF modules based on the baseline.

**Model 6:** incorporating the SIG and MSCF modules based on the baseline.

**Model 7 (Ours):** incorporating the MAF, SIG and MSCF modules based on the baseline.

**Quantitative Analysis:** Table 4 provides the quantitative results of the ablation experiments. Compared to the baseline, model 1∼3 showed JI improvements of 1.02%, 1.27%, and 1.38%, respectively, affirming the effectiveness of the MAF, SIG, and MSCF modules in enhancing segmentation performance. It is noteworthy that model 2 achieves a significant performance improvement compared to the baseline, with only a marginal increase of 0.15 M parameters and 0.11G FLOPs. This observation reflects the advantages of the SIG module in terms of computational burden and memory consumption. Furthermore, it can be observed that model 4∼7 achieves further performance enhancement by incorporating additional modules beyond model 1∼3. This observation underscores the collaborative effect of the MAF, SIG, and MSCF modules, indicating that the proposed MASDF-Net’s strong performance is attributed to the synergistic interaction among these modules.

**Figure 10:**
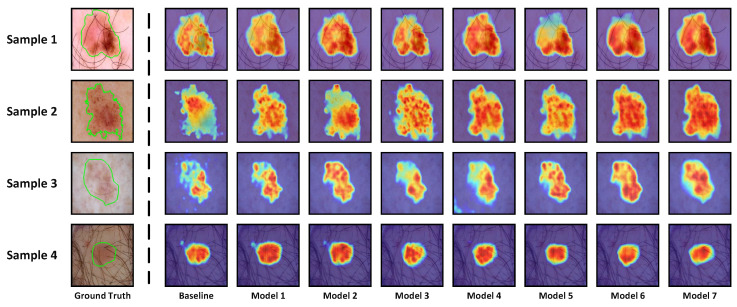
Visualization results of attention heatmaps from the last layer of the encoder. Warmer colors indicate higher attention coefficients.

**Visualization Analysis:** To provide a more interpretable conclusion, we intuitively present attention weight heatmaps generated at the last layer of the decoder in Figure 10. Samples 1, 2, and 3 depict skin lesion images with blurred boundaries. Notably, our MASDF-Net exhibits a more pronounced focus on low-contrast regions in comparison to other models. Sample 4 illustrates a skin lesion image with noise interference. It is evident that the baseline, as well as models 1 and 2, are influenced by noise, resulting in excessive attention on irrelevant areas. Conversely, the proposed MASDF-Net evidently demonstrates greater robustness.

## 5. Limitations and Future Work

Although the proposed MASDF-Net demonstrates superior performance on four public datasets, there are some potential limitations and challenges during the development and validation process, especially in terms of its application and scalability in actual clinical settings. Here are some of the issues we face and our directions for addressing them in future research:

**Computational Resource Limitations:** Due to the introduction of the PVT v2 backbone network, the proposed MASDF-Net has a large and difficult-to-reduce parameter volume. In a resource-constrained clinical environment, this could be a barrier to the deployment and operation of the model. In future research, we will attempt to replace it with other more lightweight backbone networks, and try to use model compression, optimization algorithms, and hardware acceleration technologies to reduce computational costs, making the model more suitable for use in a clinical setting.

**Data Diversity and Generalization Capability:** While the MASDF-Net performs well on specific data sets, the images of skin lesions in a clinical setting may be more diverse and complex. In future research, we will aim to validate the model’s generalization capability on a broader range of data sets and explore how techniques such as data augmentation and transfer learning can enhance the model’s ability to segment different types of skin lesions.

**Model Interpretability:** In clinical applications, doctors need to understand the decision-making process of the model. The inner workings of the MASDF-Net might not be transparent enough, which could affect the doctors’ trust and acceptance of the model. In future research, we will explore methods to enhance the interpretability of the model, such as visualizing attention maps and feature importance analysis.

**Clinical Integration:** Integrating MASDF-Net into the existing clinical workflow may encounter technical and operational challenges. This requires seamless interfacing with medical information systems, as well as compliance with medical regulations and privacy protection requirements. In future research, we will focus on how to design user-friendly interfaces and processes, as well as how to ensure data security and compliance.

## 6. Conclusions

In this study, we employ an innovative multi-attention codec network with selective and dynamic fusion (MASDF-Net) to address the several main challenges in skin lesion segmentation. The proposed MASDF-Net incorporates a PVT v2 backbone as the encoder and integrates the proposed MAF, SIG, and MSCF modules to further enhance the network’s performance, where the PVT v2 backbone network is responsible for establishing long-range dependencies among skin lesions. The MAF module combines various attention mechanisms to explore lesion areas in the deep layers of the network. The SIG module enhances the skip-connection process in traditional encoder–decoder networks, mitigating the impact of irrelevant noise like hair and artifacts in dermoscopic images. The MSCF module dynamically fuses features from different levels, enabling accurate boundary localization even in complex and blurred boundary scenarios. It’s worth noting that the modules we propose are all designed to be plug-and-play, making them easily applicable to existing encoder–decoder networks. We conduct experiments on four publicly available skin lesion datasets, and the results indicate that our model outperforms the state-of-the-art methods in performance and generalization capability. We sincerely hope that the proposed model, as a crucial component in computer-aided diagnostics, can effectively assist dermatologists in the early diagnosis and treatment of skin cancer.

## Figures and Tables

**Table 1:** Segmentation performance of different models on ISIC 2016, ISIC 2017 and ISIC 2018 dataset. The best results are in bold. The FLOPs of all models were calculated based the image in resized 224 × 224 dimensions.

Dataset	Type	Method	JI (%) ↑	DSC (%) ↑	ACC (%) ↑	SE (%) ↑	SP (%) ↑	Para (M) ↓	FLOPs (G) ↓
ISIC 2016	CNN	U-Net	84.16	90.49	95.31	92.64	95.95	34.52	50.17
		AttU-Net	84.55	90.85	95.57	93.61	95.63	34.87	51.01
		Deeplabv3+	86.29	92.17	96.19	92.30	96.54	39.76	33.19
		CE-Net	84.99	91.37	95.49	93.24	95.17	29.00	6.81
		CPFNet	84.66	91.05	95.47	92.78	96.11	30.65	6.18
		MSCA-Net	86.41	92.09	96.02	94.08	95.58	27.09	9.02
	Trans	TransFuse	86.47	92.35	96.22	**95.16**	95.39	26.17	8.83
		Swin-Unet	84.07	90.74	95.37	93.68	95.50	27.14	5.91
		UCTransNet	84.91	91.12	95.63	94.85	95.33	66.24	32.98
		Polyp-PVT	86.56	92.28	96.29	94.91	95.73	**25.10**	**4.05**
		**MASDF-Net (Ours)**	**87.35**	**92.98**	**96.68**	93.73	**96.78**	28.07	5.04
ISIC 2017	CNN	U-Net	75.60	84.19	93.07	84.16	96.78	34.52	50.17
		AttU-Net	76.04	84.40	93.06	82.74	97.50	34.87	51.01
		Deeplabv3+	76.79	84.97	93.22	83.21	**97.84**	39.76	33.19
		CE-Net	75.93	84.52	93.10	82.78	96.98	29.00	6.81
		CPFNet	76.11	84.51	93.03	83.77	96.13	30.65	6.18
		MSCA-Net	77.68	85.81	93.68	84.48	97.37	27.09	9.02
	Trans	TransFuse	78.81	86.60	94.32	85.58	97.01	26.17	8.83
		Swin-Unet	74.45	83.07	92.62	81.75	96.76	27.14	5.91
		UCTransNet	77.73	85.76	93.58	86.02	96.40	66.24	32.98
		Polyp-PVT	76.72	85.17	93.80	81.92	97.70	**25.10**	**4.05**
		**MASDF-Net (Ours)**	**80.21**	**87.65**	**94.69**	**87.80**	96.66	28.07	5.04
ISIC 2018	CNN	U-Net	81.51	88.73	95.27	90.88	96.25	34.52	50.17
		AttU-Net	81.88	89.11	95.26	92.50	95.28	34.87	51.01
		Deeplabv3+	82.59	89.56	95.93	88.98	**97.13**	39.76	33.19
		CE-Net	82.43	89.38	95.51	91.69	95.36	29.00	6.81
		CPFNet	81.23	88.41	94.81	92.47	94.64	30.65	6.18
		MSCA-Net	83.46	90.17	96.10	90.97	96.66	27.09	9.02
	Trans	TransFuse	83.63	90.37	96.12	**93.41**	95.69	26.17	8.83
		Swin-Unet	79.80	87.28	94.36	88.85	95.02	27.14	5.91
		UCTransNet	82.06	89.07	95.42	90.95	96.22	66.24	32.98
		Polyp-PVT	83.58	90.42	96.27	91.71	96.42	**25.10**	**4.05**
		**MASDF-Net (Ours)**	**84.80**	**91.22**	**96.61**	92.66	96.65	28.07	5.04

**Table 2:** Testing exclusively on challenging samples in ISIC 2018 (the values in parentheses represent the difference from the test results of all samples in ISIC 2018). The best results are in bold.

Type	Method	JI (%) ↑	DSC (%) ↑	ACC (%) ↑	SE (%) ↑	SP (%) ↑
CNN	U-Net	70.67 (−10.84)	80.98 (−7.75)	90.89 (−4.38)	88.08 (−2.80)	96.43 (+0.18)
	AttU-Net	70.74 (−11.14)	80.47 (−8.64)	91.32 (−3.94)	89.83 (−2.67)	96.45 (+1.17)
	Deeplabv3+	74.28 (−8.31)	83.10 (−6.46)	93.06 (−2.87)	88.63 (−0.35)	97.07 (−0.06)
	CE-Net	73.50 (−8.93)	82.27 (−7.11)	92.44 (−3.07)	88.33 (−3.36)	97.17 (+1.81)
	CPFNet	74.80 (−6.43)	83.63 (−4.78)	92.73 (−2.08)	90.08 (−2.39)	96.66 (+2.02)
	MSCA-Net	75.57 (−7.89)	83.86 (−6.31)	93.52 (−2.58)	89.62 (−1.35)	97.28 (+0.62)
Trans	TransFuse	75.94 (−7.69)	84.90 (−5.47)	94.60 (−1.52)	87.66 (−5.75)	97.04 (+1.35)
	Swin-Unet	74.89 (−4.91)	83.06 (−4.22)	92.85 (−1.51)	90.13 (+1.28)	97.36 (+2.34)
	UCTransNet	74.16 (−7.90)	83.58 (−5.49)	92.70 (−2.72)	89.22 (−1.73)	96.48 (+0.26)
	Polyp-PVT	75.39 (−8.19)	84.06 (−6.36)	93.56 (−2.71)	88.72 (−2.99)	96.37 (−0.05)
	**MASDF-Net (Ours)**	**79.64** (−5.16)	**87.66** (−3.56)	**97.00** (+0.39)	**90.45** (−2.21)	**98.28** (+1.63)

**Table 3:** Cross-dataset testing on ISIC 2018 and PH2 datasets, where ISIC 2018 is used as training set and PH2 is used as test set. The best results are in bold.

Type	Method	ISIC 2018→PH2				
		**JI (%) ↑**	**DSC (%) ↑**	**ACC (%) ↑**	**SE (%) ↑**	**SP (%) ↑**
CNN	U-Net	83.20	90.41	93.17	96.94	92.26
	AttU-Net	79.94	88.38	92.62	97.66	89.30
	Deeplabv3+	82.62	90.27	94.01	98.78	89.81
	CE-Net	83.81	90.73	94.38	98.05	90.53
	CPFNet	81.81	89.34	93.32	97.92	89.49
	MSCA-Net	82.68	90.21	93.81	98.20	91.21
Trans	TransFuse	83.78	90.70	94.38	**99.00**	90.52
	Swin-Unet	82.44	89.87	93.55	97.07	90.83
	UCTransNet	83.10	90.33	93.83	96.94	91.88
	Polyp-PVT	83.94	90.80	94.72	98.65	91.19
	**MASDF-Net (Ours)**	**84.64**	**91.38**	**95.06**	98.98	**91.90**

**Table 4:** Quantitative results of the ablation experiments conducted on the ISIC 2018 dataset. The best results are in bold.

Method	JI (%) ↑	DSC (%) ↑	ACC (%) ↑	SE (%) ↑	SP (%) ↑	Para (M) ↓	FLOPs (G) ↓
Baseline	82.64	89.76	96.24	91.27	96.58	**26.11**	**4.52**
Model 1	83.66	90.45	96.35	92.61	93.67	27.16	4.52
Model 2	83.91	90.55	96.38	91.42	96.72	26.26	4.63
Model 3	83.98	90.64	96.32	91.70	96.86	26.87	4.92
Model 4	84.51	90.93	96.43	92.03	96.69	27.31	4.63
Model 5	84.44	90.90	96.43	90.30	**97.71**	27.91	4.92
Model 6	84.30	90.86	96.42	91.28	97.09	27.02	5.04
**Model 7**	**84.80**	**91.22**	**96.61**	**92.66**	96.65	28.07	5.04

## Data Availability

In the context of our investigation, the experimental datasets utilized for analytical operations are accessible to the public and available for download.
